# New insights into the role of TREM2 in Alzheimer’s disease

**DOI:** 10.1186/s13024-018-0298-9

**Published:** 2018-12-20

**Authors:** Maud Gratuze, Cheryl E. G. Leyns, David M. Holtzman

**Affiliations:** 1 Department of Neurology, St. Louis, USA; 2 Hope Center for Neurological Disorders, St. Louis, USA; 3 Knight Alzheimer’s Disease Research Center, Washington University School of Medicine, St. Louis, MO 63110 USA

**Keywords:** Alzheimer’s disease, Neurodegeneration, TREM2, ApoE, Microglia, Gliosis

## Abstract

Alzheimer’s disease (AD) is the leading cause of dementia. The two histopathological markers of AD are amyloid plaques composed of the amyloid-β (Aβ) peptide, and neurofibrillary tangles of aggregated, abnormally hyperphosphorylated tau protein. The majority of AD cases are late-onset, after the age of 65, where a clear cause is still unknown. However, there are likely different multifactorial contributors including age, enviornment, biology and genetics which can increase risk for the disease. Genetic predisposition is considerable, with heritability estimates of 60–80%. Genetic factors such as rare variants of TREM2 (triggering receptor expressed on myeloid cells-2) strongly increase the risk of developing AD, confirming the role of microglia in AD pathogenesis. In the last 5 years, several studies have dissected the mechanisms by which TREM2, as well as its rare variants affect amyloid and tau pathologies and their consequences in both animal models and in human studies. In this review, we summarize increases in our understanding of the involvement of TREM2 and microglia in AD development that may open new therapeutic strategies targeting the immune system to influence AD pathogenesis.

## Background

Alzheimer's disease (AD) was first described more than a century ago by the German neuropsychiatrist, Dr. Alois Alzheimer ([1], English translation [2]), and today is the leading cause of dementia worldwide [3–5]. In the United States, deaths attributed to AD have increased by 71% between 2000 and 2013, ranking this disease as the 6th leading cause of death [6]. Although symptoms can vary greatly from one patient to another, AD results in progressive memory loss and irreversible cognitive decline. The two histopathological markers of AD are extracellular amyloid plaques composed of the amyloid-β peptide (Aβ), and neurofibrillary tangles (NFTs) within neurons derived from abnormally aggregated, hyperphosphorylated tau protein [3–5]. These defining protein aggregates are accompanied by synaptic and neuronal loss.

In addition to protein aggregation, neuroinflammatory changes are present in AD brains, including alterations in the morphology, activation and distribution of microglia and astrocytes (microgliosis and astrogliosis) as well as increased expression of inflammatory mediators [7–9]. However, the exact contributions of both microgliosis and astrogliosis in AD are not clear. While first hypothesized to contribute to AD neuropathology, gliosis and neuroinflammation seem to have more complex effects and could be either beneficial or damaging in those with AD (for review [10]). For example, reactive microglia and astrocytes can contribute to the clearance of Aβ [11–13]. Conversely, the production of pro-inflammatory cytokines like TNFα (Tumor necrosis factor α) or IL1-β (Interleukin 1β) resulting from glial activation are harmful and toxic to neurons (for review [14, 15]). Many studies also suggest that neuroinflammation exacerbates tau phosphorylation [16–18]. Altogether, these data suggest the possibility that gliosis and neuroinflammation have neuroprotective roles early in AD by controlling amyloid load, but later can become toxic to neurons and act as a catalyst for neurodegeneration.

Recent genomic studies have identified several novel genetic risk factors linking neuroinflammation and AD. Highly penetrant mutations in *APP*, *PSEN1*, and *PSEN2* are known to cause rare, autosomal dominant AD, where individuals develop the disease in mid-life [19]. However, the majority of AD cases are sporadic and late-onset, the causes of which are still unknown. Late-onset AD (LOAD) seems to be multifactorial, with age, environmental, and genetic factors contributing to disease risk, manifestation and progression. Interestingly, the genetic predisposition in LOAD patients is considerable, with a heritability estimate of 60–80% [20]. The most common genetic risk factor is the apolipoprotein E (ApoE) gene (for review [21]). *APOE* is encoded by three common alleles: ε2, ε3, and ε4. One copy of the ε4 allele of APOE increases LOAD risk approximately 3-4 fold while two ε4 copies increases LOAD risk by as much as 12-fold [22, 23]. Interestingly, the ε2 allele is associated with a decreased risk for LOAD and a later onset of disease. There are several mechanisms by which ApoE appears to play a role in AD pathogenesis. One important effect is that ApoE isoforms influence Aβ clearance, aggregation and metabolism [24–26]. In addition, recent studies suggest that ApoE modulates tau-mediated neurodegeneration in an isoform-specific manner [27].

For the last ten years, new whole-genome sequencing studies and genome wide association studies (GWAS) have made it possible to highlight several novel genetic factors linked with increased risk of LOAD. Several of these genetic risk factors encode proteins involved in microglial function and inflammation including TREM2, CD33, CR1, ABCA7 and SHIP1 (for reviews [28, 29]). In this review, we summarize the recent explosion of studies aiming to understand the role of microglia in LOAD. In particular, we focus on TREM2, a receptor of the innate immune system expressed in several types, as variants in the *TREM2* gene have been found to increase LOAD-risk by 2-4 fold, similarly to what has been observed in patients with one copy of APOE ε4. Many new models have been created to better understand the contributions of TREM2 in LOAD in light of this finding. Thus far, TREM2 studies further reiterate both the beneficial and detrimental effects of gliosis on neuronal health and degeneration, which are dependent on the context of the pathological insult and stage of disease. We further explore potential mechanisms by which TREM2 signaling may alter LOAD neuropathology. A better understanding of TREM2 and its impact in the disease is critical as TREM2 is currently being explored as a therapeutic target in LOAD.

### 1) TREM2 structure and expression

TREM2 belongs to a family of receptors referred to as the triggering receptors expressed on myeloid cells (TREM). Members of the TREM family are cell surface transmembrane glycoproteins with V-immunoglobulin extra-cellular domains and cytoplasmic tails [30]. The TREM2 gene is located on human chromosome 6p21 and encodes a 230-amino acid glycoprotein [31]. The TREM2 gene is expressed in a subgroup of myeloid cells including dendritic cells, granulocytes, and tissue-specific macrophages like osteoclasts, Kuppfer cells and alveolar macrophages [32–38]. In the brain, TREM2 is exclusively expressed by microglia [39–44]; however, there is some discordance regarding the level of its expression//translation [45–47] and whether or not TREM2 is present in all or only a subgroup of microglia [48] in mice and humans. Interestingly, the expression of TREM2 varies depending on the particular region of the central nervous system (CNS) [39, 49], with a higher expression in the hippocampus, the spinal cord and the white matter [41]. Its expression is modulated by inflammation, although inflammatory effects appear to be opposite *in vitro* and *in vivo*. Expression of anti-inflammatory molecules enhances TREM2 expression [50] while expression of pro-inflammatory molecules, such as TNFα, IL1β or lipopolysaccharide (LPS), decrease TREM2 expression *in vitro* [32, 51, 52]. TREM2 expression is up regulated in pathological conditions such as Parkinson’s disease (PD) [53], Amyotrophic lateral sclerosis (ALS) [54], stroke [55], traumatic brain injury [56] and AD [47, 57–59]. In AD, increased expression of TREM2 has been confirmed in patients [47, 57–59] and in mouse models of amyloid and tau pathology [45, 60–63] and seems to be associated with the recruitment of microglia to amyloid plaques [59, 64]. Interestingly, aging is also a factor that increases TREM2 expression in both mice and humans [41, 60]. We could speculate that the acute inflammation mimicked by *in vitro* studies first induces a decrease of TREM2 expression while chronic inflammation observed in pathological conditions, such as AD, results in an increase of TREM2 expression.

### 2) TREM2 signaling and ligands

**Fig. 1:**
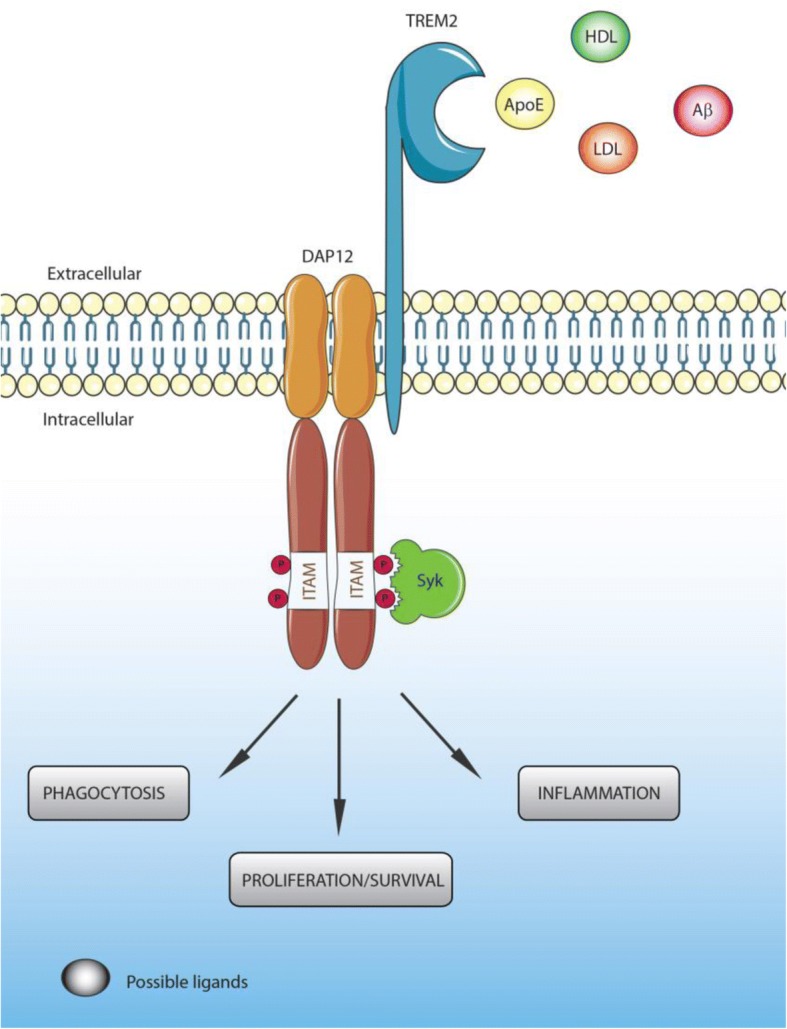
TREM2 ligands, signaling and functions**.** Ligands binding to TREM2 induce the association of TREM2 to DAP12 through an electrostatic interaction between a conserved positively-charged lysine in TREM2 (aa186) and a negatively- charged aspartic acid residue in DAP12, generating tyrosine phosphorylation of DAP12 within its immunoreceptor tyrosine-based activation motifs (ITAMS) by Src family kinases

TREM2 acts principally through the intracellular adaptor DAP12 (DNAX-activation protein 12, also known as TYROBP) through its short cytoplasmic tail [32, 65, 66]. Indeed, ligand-bound TREM2 is incapable of initiating intracellular signaling without DAP12 [43]. The association of TREM2 with DAP12 is coordinated by an electrostatic interaction between a conserved positively-charged lysine in TREM2 (aa186) and a negatively- charged aspartic acid residue in DAP12 (Figure 1) [66, 67]. TREM2 ligation generates tyrosine phosphorylation of DAP12 within its immunoreceptor tyrosine-based activation motifs (ITAMS) by Src family kinases (Figure 1). This phosphorylation creates a docking site for the SH2 domains of several molecules, initiating a signaling cascade and subsequent immune response [66]. The principal kinase recruited by the ITAM region of DAP12 is Syk, which activates downstream signaling components including phosphatidylinositol 3-kinase (PI3K), Akt, mitogen-activated protein kinases (MAPK) and increases intracellular calcium levels [43, 68–70]. TREM2 can also act through the DAP-10 adaptor, a relative of DAP-12, allowing the recruitment of PI3K [71].

The exact identities of ligands that activate TREM2 remain uncertain. Early studies found that the TREM2 extracellular domain is able to bind microbial products such as LPS or lipoteichoic acids (LTA) [72]. Similarly to other TREM family members [73], lipids can bind and activate TREM2. Indeed, the putative structure of TREM2 contains a potential phospholipid binding site [73], which is confirmed by its crystal structure [74]. In addition, several studies demonstrated binding between TREM2 and lipids from cell membranes and lipoprotein complexes [73, 75, 76]. TREM2 has been reported to bind high-density lipoproteins (HDL), low-density lipoproteins (LDL) and several apolipoproteins such as ApoA1, ApoA2, ApoB and clusterin (ApoJ) [76–79]. However, one of the most well documented ligands of TREM2 is ApoE [76, 78, 79]. *In vitro* studies have demonstrated that lipidation of ApoE is not required for TREM2-ApoE binding [76, 78–80], although Yeh et al. suggested that ApoE lipidation enhanced this interaction [76].

It is interesting to note the interaction between TREM2, ApoE, and clusterin because all three are important genetic risk factors for LOAD, although binding between TREM2 and ApoE occurs with similar affinities between the three different AD-associated ApoE isoforms [64, 78, 79]. In addition, a recent paper characterized Aβ as a ligand of TREM2 [81]. The authors found that Aβ was able to directly bind to TREM2 and activate TREM2 signaling through DAP12 *in vitro*. Interestingly, an immunoprecipitation assay demonstrated a strong enhancement of TREM2-Aβ interaction with oligomeric forms of Aβ compared to monomers [81]. While most ligands of TREM2 have been identified *in vitro,* a recent *in vivo* study by Ulrich et al. strongly argues that ApoE and TREM2 are in the same pathway [82]. Given that a lack of TREM2 expression impairs plaque-associated microgliosis in amyloid models, Ulrich et al. tested whether a lack of ApoE expression similarly affected the microglial response to amyloid plaques. They observed that mice lacking ApoE phenocopied mice lacking TREM2 in regards to the plaque-associated microglial response.

### 3) TREM2 functions

While the identities of TREM2 ligands remain uncertain, several functions of TREM2 have been well characterized in the last decade. TREM2 enhances the rate of phagocytosis. *In vitro*, loss of TREM2 in microglia and macrophages results in decreased phagocytosis of apoptotic neurons [43, 55, 78, 83], cellular debris [43] and bacteria or bacterial products [84–87]; increasing TREM2 expression improved phagocytosis rate of these substrates [43, 63, 85, 87, 88]. Moreover, TREM2 has been associated with Aβ uptake *in vitro*, which will be discussed in a following section. *In vivo*, TREM2 KO mice had decreased levels of activated microglia and phagocytes in an experimental stroke model [55]. In a mouse model of multiple sclerosis, TREM2-transduced myeloid cells with lentivirus showed increased lysosomal and phagocytic activity [88]. It is noteworthy that lentiviruses and adeno-associated viruses **(**AAVs) have not been shown to transduce these cells in the brain efficiently. Regarding Aβ, microglia from 5XFAD mice without TREM2 internalized less methoxy-×04 (indicative of fibrillar Aβ) compared to microglia from 5XFAD mice with TREM2 [89]. In the same mouse model, Aβ content was significantly reduced within CD68-immunolabeled microglial phagosomes when TREM2 was absent [90]. These findings in TREM2 deficient mice from multiple pathological models corroborate *in vitro* studies regarding TREM2’s role in phagocytosis.

TREM2 also modulates inflammatory signaling. Most studies agree on the anti-inflammatory properties of TREM2. Indeed, Toll-like receptor (TLR) stimulation induced a higher release of pro-inflammatory cytokines, including TNFα and IL6, by bone marrow-derived macrophages lacking TREM2 [50] or DAP12 [91]. Anti-inflammatory effects of TREM2 after TLR stimulation have been confirmed in several cell lines [88, 92, 93]. In microglia, the knockdown of TREM2 signaling increased TNFα and NO synthase-2 transcription (NOS2), while overexpression of TREM2 decreased gene transcription of TNFα, IL1β and NOS2 [43]. TREM2 also appears to signal through anti-inflammatory pathways in several pathological mouse models [55, 88, 94]. A recent study demonstrated that TREM2 mediates the switch from a homeostatic to a neurodegenerative microglia phenotype in APPPS1 and SOD1 (ALS mouse model) mice by inducing APOE signaling, a negative regulator of the homeostatic microglia transcription program [95]. Conversely, some studies reported that TREM2 promoted pro-inflammatory signaling [32, 96]. Inspection of genes with the highest connectivity to TREM2 revealed both anti- and pro-inflammatory gene clusters in the brain [41]. These last findings strongly suggest a more complex action of TREM2 on inflammatory processes.

TREM2 has also been shown to modulate myeloid cell number, proliferation and survival. The ability of TREM2 to influence and, more specifically, to increase the number of myeloid cells is mostly described in disease contexts [55, 56, 84]. TREM2 enhances proliferation of several myeloid cell populations including microglia. *In vitro*, reduction of TREM2 in primary microglia cultures resulted in cell cycle arrest at the G1/S checkpoint [97]. Similarly*,* a decrease of microglial proliferation has been observed in different disease models when deficient for TREM2 [89, 98]. Finally, several studies suggested TREM2 as a key factor for myeloid cell survival. Indeed, Zheng et al. reported decreased survival of primary microglia and BV2 microglial cells along with an alteration of the Wnt/β-catenin activation pathway when TREM2 expression was reduced [97]. In accordance with these data, bone marrow derived macrophages and microglia deficient for TREM2 showed a lower survival rate after CSF1 starvation [75, 77, 99]. On the other hand, TREM2 activation improved dendritic cell survival through activation of the ERK pathway [32].

The functions of TREM2 previously described demonstrate the importance of TREM2 at the physiological and pathological level. Somehow, the loss of these functions in humans with homozygous loss-of-function mutations in TREM2 suffer from a severe form of dementia with bone cystic lesions known as Nasu-Hakola disease [100, 101]. How and why this particular disease occurs due to loss of TREM2 function is not yet clear. Several TREM2 variants in the human population are able to impair but not block functional TREM2 signaling and impact the onset and progression of AD as will be described in an upcoming section of this review. It is noteworthy that studies have also associated some TREM2 variants with other neurodegenerative diseases such as ALS [54, 102], Parkinson’s disease [102, 103] and frontotemporal dementia [104–106], although these observations are still somewhat controversial [102, 104, 107].

### 4) TREM2 variants and Alzheimer’s disease

Thanks to the recent development of whole genome sequencing and genome-wide association studies (GWAS), several genetic variants have been identified that increase the risk of developing LOAD. Among them, several rare variants in TREM2 have emerged that significantly increase LOAD risk by 2- to 4- fold, comparable to the increased risk associated with having one copy of APOE ε4. The most common and most well studied TREM2 variant known to increase the risk of AD is rs75932628, a single nucleotide polymorphism encoding an arginine-to-histidine missense substitution at amino acid 47 (R47H). The R47H variant was first identified as a risk factor for LOAD in 2013 by two independent studies on subjects of people of European or North American descent [108] and Icelandic subjects [109]. The association between the R47H variant and LOAD in populations of European descent was thereafter corroborated by several studies [104, 107, 110–118]. However, this risk variant has mostly present in European populations and the association of the R47H mutant with increased LOAD risk does not seem to exist in Chinese and African-American populations [119–123]. LOAD patients with the R47H variant display an earlier onset of symptoms [107, 109] and faster cognitive decline [124] compared to non-carriers, although these results are not always consistent between studies [107, 117, 125]. Interestingly, in a European cohort, the R47H variant of TREM2 increased the level of total tau protein in the CSF without affecting Aβ42 [102]. These data suggest a link between AD-associated pathologies and the R47H TREM2 variant. Other TREM2 variants have also been suggested as risk factors for developing LOAD, including R62H (rs143332484), D87N (rs142232675), T96K (rs2234353), L211P (rs2234256) and R136Q (rs149622783) [77, 108, 110, 112, 115, 126].

Identification of these new TREM2 variants as LOAD genetic risk factors has prompted many scientists to study their impact on TREM2 functions. Most of the studies have determined that AD-associated TREM2 variants do not affect folding, expression, stability or structure of TREM2 in AD brains [58, 77, 85]. However, Kober et al. do suggest that the R47H mutation induces a small, but measurable, conformational change in TREM2, and that the R47H and R62H TREM2 variants exhibited slightly decreased stability compared to the common variant [74]. AD-associated variants do appear to affect the affinity of TREM2 for its ligands. Indeed, binding assays revealed that R47H, R62H and D87N variants exhibit impaired interactions with lipoprotein ligands including ApoE, LDL and clusterin *in vitro* [74, 76, 78, 79]. Conversely, Kober et al. reported that the T96K variant increased TREM2 affinity to cell-surface ligands, while confirming reduced binding of cell-surface ligands by R47H [74]. AD-associated variants disturb TREM2 signaling in a pattern similar to that observed for affinity: R47H and R62H negatively affect TREM2 activity *in vitro*, while T96K activity was enhanced compared to the common variant of TREM2 [75, 77]. However, this correlation between affinity and signaling strength does not apply to the D87N variant, which induces a decrease in ligand binding but an increase in TREM2 signaling [77]. This highlights the need for better understanding of the complex action of TREM2. Finally, R47H and R62H variants have been shown to slightly alter phagocytic functions of TREM2 *in vitro* [76, 85]. Moreover, R47H missense mutation impairs TREM2 maturation and alters shedding by α-secretase [85]. Finally, Yeh et al. demonstrated a decrease of Aβ-lipoprotein complex uptake by blood monocyte-derived macrophages from patients heterozygous for the R62H variant [76].

Over the last 5 years, considerable efforts have been made to better understand how different TREM2 variants can affect the risk of AD *in vitro*. These studies have found that these variants result in a decrease of TREM2 functions. It is now essential to confirm and further explore these data *in vivo* using animal models of AD and in patients in order to elucidate the precise mechanisms linking the TREM2 variants to AD.

### 5) TREM2 and amyloid pathology in AD

#### TREM2 and amyloid burden

A large majority of *in vivo* studies aimed at understanding the link between TREM2 and AD have focused on amyloid pathology. Most of these studies used different mouse models of amyloid deposition with total or partial deletions of TREM2. Unfortunately, while *in vitro* studies suggested that TREM2 is strongly involved in Aβ40 and Aβ42 uptake by microglia [63, 85, 127], experiments performed *in vivo* found more inconsistent results regarding TREM2 function in Aβ uptake by microglia. The APPPS1-21 [128] and 5xFAD [129] mouse models have mainly been used to assess the effect of TREM2 deficiency or haplo-insufficiency on Aβ accumulation. In APPPS1-21 mice, TREM2 haplo-insufficiency did not affect Aβ accumulation in the cortex [130]. A total deletion of TREM2 in the APPPS1-21 mouse model resulted in a decreased Aβ burden in the cortex of 2-month-old mice but in a higher Aβ accumulation in the cortex of 8-month-old mice [131]. In 5xFAD mice, Wang et al. reported that TREM2 deletion increased Aβ pathology (insoluble Aβ42 and Aβ load) in the hippocampus but not in the cortex of 8.5-month-old mice, with an intermediate phenotype in TREM2-haplo-insufficient mice [75, 89]. These data suggest an age-dependent or Aβ burden-dependent effect of TREM2 on Aβ deposition. Therefore, TREM2 could result in greater Aβ deposition in the early stages of the disease and then result in less Aβ deposition in later stages. A time course analysis of Aβ burden in these two TREM2-deficient models will be necessary to confirm this hypothesis. Moreover, the use of a less aggressive model of Aβ deposition will be useful in order to finely dissect the effect of TREM2 on Aβ accumulation. [45, 75, 130] [132, 133]

#### TREM2 and microglial function

Whereas the effect of TREM2 on Aβ deposition remains unclear, studies have unanimously reported decreased microglial activation in APPPS1-21 and 5xFAD mice deficient or haplo-insufficient for TREM2, resulting in a subsequent reduction of plaque-associated microglia [45, 75, 130]. Importantly, microglia also failed to cluster around plaques in APPPS1-21 mice haplo-insufficient or deficient for DAP12 [90], confirming the need for TREM2 signaling to activate microglia and recruit them to the plaques. The inability of microglia to cluster around plaques without TREM2 was associated with defects in plaque compaction, microglia proliferation, and increased levels of dystrophic neurons [75, 89, 90]. Moreover, microglia that lacked TREM2 exhibited strong metabolic defects, including low ATP levels and elevated stress markers such as autophagic vesicles. This was caused by defective mammalian Target Of Rapamycin (mTOR) signaling [134]. These results suggest that TREM2 provides trophic support for microglia in stressful conditions. In cases of prolonged stress or activation, as seen in AD, a defective TREM2 could then alter microglial functions and survival through deficient mTOR signaling, resulting in exacerbation of AD neuropathologies.

Recently, Lee at al. used a BAC transgene to induce human TREM2 (hTREM2) expression in 5xFAD mice [135]. Compared to 5xFAD mice deficient or haplo-insufficient for TREM2 [75, 89, 136], 5xFAD mice expressing human TREM2 exhibited reduced insoluble and soluble Aβ42, diminished plaque area, more compact plaques and fewer dystrophic neurites in the cortex [135]. The authors demonstrated that expression of hTREM2 resulted in a reprogrammed microglial gene expression signature. Interestingly, human TREM2 expression was associated with the upregulation of some “disease-associated microglial genes” involved in microglial function, such as phagocytosis. Following this observation, the authors confirmed that phagocytic microglia markers such as CD68 and Lgasl3 in were increased in 5xFAD mice expressing hTREM2. This study confirmed several TREM2 functions previously described in TREM2-deletion mouse models and suggested that reprogrammed microglial gene expression is a key function of TREM2 in the Aβ-deposition models.

Recent findings by Zhao et al. revealed that TREM2 is an Aβ receptor that mediates microglial functions [81, 137]. As previously mentioned, the authors demonstrated TREM2-Aβ interactions using co-immunoprecipitation, cell-free, solid-phase and cell-based binding assay; this interaction was stronger with oligomeric forms of Aβ compared to monomers [81, 137]. Aβ/TREM2 binding activated TREM2 signaling and induced microglia depolarization and Aβ degradation. Moreover, microglia without TREM2 displayed defective clearance of Aβ, with longer internalization of Aβ and an impaired Aβ-induced pro-inflammatory response. *In vivo*, Zhao et al. described alterations in Aβ degradation as well as microglial proliferation and apoptosis in Aβ-injected TREM2 deficient mice [81]. Altogether, these data suggest that oligomeric forms of Aβ may activate TREM2 signaling through direct binding resulting in a pro-inflammatory response, Aβ degradation and microglial proliferation. Moreover, these results could explain why TREM2 is necessary for the recruitment of microglia to plaques.

Importantly, Zhao et al. also reported that the R47H and R62H hTREM2 variants compromised the interaction between oligomeric Aβ and TREM2-Fc [81]. We can therefore speculate that the mechanism by which TREM2 variants increase the risk of LOAD is by altering TREM2’s Aβ-receptor functions. However, these *in vitro* data have not yet been confirmed by *in vivo* studies [82]. Indeed, Ulrich et al. demonstrated that a lack of ApoE expression affects microglial recruitment to amyloid plaques, which is phenotypically similar to what has been observed in mice lacking TREM2 expression. These *in vivo* observations suggest that if TREM2 is able to bind Aβ, as suggested by *in vitro* studies, it does not seem able to bind Aβ in plaques in the absence of ApoE to result in microglial recruitment and reduce neuritic dystrophy. Further studies characterizing AD-associated TREM2 variants in the context of amyloid pathology are thus needed to confirm this hypothesis and better understand how TREM2 variants promote LOAD.

#### The biologic impacts of hTREM2 variants

Two studies aimed at clarifying the impact of human TREM2 variants in LOAD have been published to date. In the first of these studies, Song et al. generated transgenic mice expressing the common variant or R47H variant of human TREM2 *via* BAC transgenes in a TREM2-deficient 5xFAD mouse background [132]. They reported that only the common variant of TREM2 was able to restore microgliosis and microglial activation induced by amyloid pathology in this model, while mice expressing the R47H variant displayed impaired microglial activation and recruitment to plaques. These results are similar to the observations made in TREM2-deficient 5xFAD mice [132]. Moreover, the authors found that soluble TREM2 released from microglia membranes is found in plaques and neurons of 5xFAD mice with the common variant of TREM2, but not in 5xFAD mice with the R47H variant of TREM2. Importantly, soluble TREM2 (corresponding to the TREM2 ectodomain previously described) was identified as a receptor for oligomeric Aβ [81, 137]. This suggests a possible direct binding between soluble TREM2 and plaques in the brains of 5xFAD mice expressing the common variant of TREM2 but not in the brains of mice expressing the R47H variant [81, 137]. However, the impact of this possible interaction between soluble TREM2 and oligomeric Aβ on AD remains to be further explored.

In a subsequent study, Cheng-Hathaway et al. used APPPS1-21 mice in which CRISPR/Cas9 was used to knock in the R47H TREM2 variant into the endogenous mouse TREM2 gene [133]. APPPS1-21 mice heterozygous for the R47H TREM2 variant exhibited reduced association of microglia to plaques, lowered Aβ-induced microglial activation and proliferation, more diffuse amyloid plaques and increased plaque-associated neuritic dystrophy. These results are comparable to observations made in both Aβ-deposition and APPPS1-21 mouse models that are haplo-insufficient or deficient for TREM2 [45, 75, 89, 130, 131]. Surprisingly, a strong reduction of TREM2 mRNA was observed in APPPS1-21 mice heterozygous for the R47H TREM2 variant, which differs from a human study that reported no change in TREM2 expression in the brain of AD patients heterozygous for the R47H variant [58]. Interestingly, a similar decrease of plaque-associated microglia, the presence of less compact plaques and higher neuritic dystrophy around plaques has also been observed in AD patients expressing the R47H variant of TREM2 compared to those patients expressing the common TREM2 variant, confirming the partial loss-of-function caused by the R47H variant [136]. Taken together, these *in vivo* studies confirmed the suspected partial TREM2 loss-of-function phenotype of the LOAD-associated R47H variant. Importantly, a new study by Xiang et al. has explored the cause of TREM2 RNA reduction in this CRISP/Cas9 model [138]. They reported that the R47H variant activated a cryptic splice site, which introduced a premature stop codon in mice but not in human TREM2, resulting in haplo-insufficiency of the Cheng-Hathaway et al. model. These data strongly suggest that results obtained with this model should be interpreted carefully and may not be directly translatable to humans.

To conclude, these studies in mouse models of amyloid deposition indicate a critical function for TREM2 in the clustering of microglia around plaques, plaque compaction, and microglia proliferation and activation, which may be disturbed by some rare TREM2 variants. TREM2 signaling appears to both positively and negatively affect amyloid pathology depending on disease progression. AD-associated TREM2 variants may induce partial loss-of-function phenotypes, resulting in an inability of microglia to cluster around plaques. These findings advance our understanding of TREM2 involvement in the response of microglia to Aβ aggregation and its consequences and strongly encourage targeting and enhancing TREM2 expression and/or signaling early in AD pathogenesis to reduce Aβ-induced brain injury associated with TREM2 defects in AD. However, the Aβ deposition phase of AD occurs predominantly prior to symptom onset in humans. Questions still remain regarding the role of TREM2 and its different variants in later stages of AD, in particular in tau pathology and tau seeding.

### 6) TREM2 and tau pathology in AD

#### First clues linking TREM2 to tau pathology

Numerous findings in the literature suggest a link between tau pathology and TREM2 in LOAD. Indeed, in the cerebrospinal fluid (CSF) of AD patients, soluble TREM2 has been shown to correlate with total and phosphorylated tau (Thr181) levels, but not with levels of Aβ42 [139]. Moreover, AD patients harboring the R47H variant of TREM2 display higher levels of both total tau and phosphorylated tau (Thr181) in CSF compared to non-carriers, without any change in Aβ42 levels [102, 140]. Importantly, levels of phosphorylated tau in the CSF correlate with tau pathology burden in the brain (both in terms of neurofibrillary tangles and hyperphosphorylated tau loading) [141] and with neuronal loss and cognitive decline in AD patients [141–143]. Importantly, a study reported increased tau hyperphosphorylation and axonal dystrophy around amyloid plaques in humans harboring the R47H variant of TREM2 [90]. A study also found a positive correlation between TREM2 mRNA levels and tau burden in a cohort of 20 AD patients and 12 controls [57]. Additionally, microglia have been strongly characterized as key players in tau pathology and propagation [144–147]. Indeed, in a mouse model of tau propagation using an injection of AAV2/6-SYN1 promoter driving the expression of human P301L tau 1–441 mutant into the entorhinal cortex, Asai et al. report that depleting microglia dramatically suppressed the propagation of Tau [147]. Moreover, Luo et al. demonstrated that microglia degrade human tau species released from AD brains and eliminate NFTs from PS19 mice, a mouse model of tauopathy harboring the P301S human tau mutation [145]. In hTau mice, another tauopathy mouse model, microglial activation has been shown to correlate with a deficit in spatial memory and the spread of tau pathology [146]. While preliminary, these data suggest a role for microglia in tau pathology and tau propagation in AD, which can be affected by AD-associated TREM2 variants. However, only a few studies have investigated the link between tau and TREM2 compared to the extensive number of studies aimed at understanding the link between TREM2 and Aβ.

Some studies have assessed tau phosphorylation in Aβ-deposition mouse models deficient for TREM2. However, the results reported in these studies are inconsistent, and demonstrate either an increase [89] or decrease [45] of phosphorylated tau in mice deficient for TREM2. This may be because of differences in the mouse models used (5xFAD *vs.* APPPS1). A recent study also suggested that overexpression of TREM2 through ICV injection of AAV encoding the murine TREM2 gene was able to decrease surgery-induced tau hyperphosphorylation in APPswe/PS1dE9 mice [148]. However, it is important to note that robust microglial transduction has not been reported *in vitro* using commonly used AAVs (1-9).

#### TREM2 functions in models of pure tau pathology

Although investigating the effects of TREM2 on tau pathology in the context of amyloid pathology is essential to better understand AD, studies of the direct link between TREM2 and tau pathology are very rare. Only 5 studies of TREM2 in pure tau pathology exist today in the literature. Two of these studies have been done by Jiang et al. in PS19 mice, a model of tauopathy harboring the P301S mutation [149, 150]. In the first study, the authors suggested that the silencing of brain TREM2 *via* injection of a lentivirus containing TREM2 shRNA was able to exacerbate tau phosphorylation through neuroinflammation-induced hyperactivation of tau kinases [150]. Moreover, data presented in this study suggests an exacerbation of neurodegeneration and higher spatial learning deficits in PS19 mice expressing TREM2 shRNA compared to mice not deficient in TREM2. In the second study, they induced TREM2 overexpression in microglia of PS19 mice with a lentivirus containing TREM2 cDNA. In agreement with their first study, the authors observed that overexpression of TREM2 in microglia reduced neurodegeneration, spatial cognitive impairments and tau hyperphosphorylation through the suppression of neuroinflammation-induced hyperactivation of tau kinases [149]. Although both articles have relevant hypotheses and provide encouraging results, a significant technical issue exists in these studies. Clear evidence that the lentiviruses utilized were able to infect and alter TREM2 levels in microglia is lacking. In the first study, the authors were able to confirm an increase in TREM2 mRNA levels specifically in microglia during disease progression in PS19 mice [150]. However, they did not use the same technique to assess the efficiency of their lentiviruses and only assessed TREM2 mRNA levels in whole cortex and hippocampus (not only in microglia) [149, 150]. To confirm that the lentiviruses altered TREM2 expression in microglia, they performed TREM2 immunofluorescence staining in mouse brains but the authors mention possible non-specific binding with the antibodies utilized [149, 150]. To summarize, due to the lack of important controls, results obtained in these studies are not sufficient to confidently understand how TREM2 affects tau pathology.

Three recent studies tackled this problem by crossing TREM2 knockout mice with two different murine models of tauopathies [151–153]. In the first study, Leyns et al. reported a decrease in neurodegeneration as well as attenuated microgliosis and astrogliosis in the brains of PS19 mice deficient for TREM2 [152]. Interestingly, no differences were observed for tau phosphorylation and insolubility in PS19 mice with or without TREM2. These unexpected results suggest that TREM2 promotes neuroinflammation and neurodegeneration in the context of tauopathy. In a second study, Sayed et al. [153] found that TREM2 haplo-insufficiency, but not complete loss of TREM2, increased tau pathology. Further, whereas complete TREM2 deficiency protected against tau-mediated microglial activation and atrophy as seen by Leyns et al. [152], TREM2 haplo-insufficiency elevated expression of proinflammatory markers and exacerbated atrophy at a late stage of disease. Taken together, these 2 studies suggest that partial or normal TREM2 function contributes to tauopathy as well as tau-mediated damage and that complete loss of function also decreases tau-mediated brain injury. In a third study, Bemiller et al. crossed TREM2 knockout mice with hTau mice [151], which is a mouse model of tauopathy expressing all human Tau isoforms in a murine tau knockout background [154]. Bemiller at al. confirmed the decrease of microgliosis in TREM2-deficient hTau mice, as previously seen in TREM2-deficient PS19 mice [151–153]. However, unlike studies on PS19 mice, complete deletion of TREM2 in hTau mice exacerbated tau phosphorylation and insolubility. The authors suggest that these changes are driven by the activation of stress-related tau protein kinases in TREM2-deficient hTau mice. Neurodegeneration was not evaluated in this study. These pure tauopathy mouse models suggest a complex relationship between TREM2 and Tau pathology, which requires further research. hTau and PS19 mice are independent mouse models and develop distinctive pathologies, which may explain differences between these models. Given the results of the current studies, it will be important to understand the effects of human TREM2 and human TREM2 variants in both pure tauopathy models that develop tau pathology and neurodegeneration as well as the effects of TREM2 in models that develop both Aβ and tau pathology.

Recently, an interesting study evaluated molecular and pathological interactions between Aβ42, tau, TREM2, and DAP12 in *Drosophila* models [155]. They created flies that expressed human tau in photoreceptor neurons and either WT or R47H TREM2/DAP12 complexes in glia cells simultaneously. Results obtained in this new model demonstrated that glial expression of both TREM2^WT^/DAP12 and TREM2^R47H^/TDAP12 complexes significantly exacerbated tau-mediated neurodegeneration without affecting tau phosphorylation and insolubility, in agreement with the Leyns et al. and Sayed et al. studies in TREM2 deficient PS19 mice [152, 153]. On the other hand, a recent *in vitro* study using a microglia/neuron co-culture model, reported that depletion of TREM2 exacerbated tau phosphorylation *via* an increase in the microglial inflammatory response [156]. These results are in agreement with the findings reported by Bemiller et al.

Taken together, studies assessing the link between tau pathology and TREM2 suggest a biphasic effect of TREM2 loss-of-function, similar to what has been seen for amyloid pathology. In the early stages of the disease, dysfunctional TREM2 can promote tau pathology (both hyperphosphorylation and aggregation), while the complete loss of TREM2 function in advanced stages of the disease seems to protect from neurodegeneration.

### 7) TREM2 and ApoE: a close partnership in AD pathogenesis?

Beyond the direct contribution of TREM2 and its AD-related variants on the two histopathological markers of LOAD (i.e. amyloid plaques and NFTs), a new hypothesis has started to emerge suggesting a collaboration between ApoE and TREM2 in LOAD pathogenesis. This hypothesis is based on a combination of several observations. First, APOE and TREM2 are, to date, the two largest genetic risk factors that influence the development of LOAD [157]. Second, ApoE is now well-characterized as a TREM2 ligand *in vitro,* which may stimulate TREM2 functions [76, 78–80]. Interestingly, binding between TREM2 and ApoE occurs to a similar extent between the three different ApoE isoforms [64, 78–80]. Another study showed higher TREM2 expression in patient-derived mononuclear blood cells from ApoE ε4-carriers with mild cognitive impairment and AD compared to non-carriers [158]. Moreover, decrease of TREM2 in microglia has been reported in mice expressing ApoE ε4 [159]. Recently, Murray et al. suggested that ApoE ε4 is required in TREM2 R47H variant-carriers for AD to develop, although larger cohorts and statistical analyses are needed to support this hypothesis [160].

At the microglial level, quantification of ApoE transcripts isolated from both WT and TREM2-deleted mice using fluorescence-activated cell sorting and nanostring technology revealed a downregulation of ApoE expression in TREM2-deleted mice [161]. In PS19 mice, Leyns et al. reported the accumulation of ApoE-positive puncta specifically in microglia [152]. Deletion of TREM2 in this model strongly lowered the number of microglia containing ApoE puncta, and decreased the gene expression of ApoE in the cortex. Moreover, the same reduction in neurodegeneration induced by tau pathology in PS19 mice has been reported when mice lack TREM2 [152] or ApoE [27]. This suggests a strong contribution of TREM2 and ApoE in neurodegeneration in this model. Recent findings by Ulrich et al. also suggest that ApoE and TREM2 are in the same pathway [82]. Indeed, amyloid mice lacking ApoE phenocopied mice lacking TREM2 in regards to the plaque-associated microglial response. Taken together, these data suggest a possible relationship between TREM2 and ApoE in AD, although the exact nature of this alliance and its consequences in AD remained, until recently, poorly understood.

A new study has shed light on the function of the TREM2/ApoE connection in neurodegenerative diseases including AD [95]. In this study by Kraesmann et al., a specific molecular signature has was identified in microglia from several mouse models of neurodegenerative diseases, including AD [95]. This neurodegeneration-associated phenotype acquired by microglia (MGnD) is characterized by transcriptional changes, including decreased expression of 68 homeostatic genes, and increased expression of 28 inflammatory genes. Because the downregulation of ApoE observed in microglia during development is correlated with a homeostatic profile, the authors next aimed to assess the role of ApoE in the induction of MGnD in microglia [162]. To this end, they performed transcriptomic analysis in APOE-deleted microglia and observed that ApoE regulates the MGnD transcriptional program. Moreover, because TREM2 binds ApoE, the authors then evaluated if the ApoE-induced switch from a homeostatic profile to MGnD in microglia was TREM2-dependent. Deletion of TREM2 in APPPS1-21 mice suppressed the MGnD profile, locking microglia in the homeostatic phenotype. This suggests that the TREM2/ApoE pathway is able to drive the switch from homeostatic microglia to neurodegenerative microglia, MGnD being less effective at preventing neuronal loss. This novel study provides many answers regarding the relationship between TREM2 and ApoE in neurodegenerative diseases. However, it is still not clear whether ApoE or TREM2 are upstream or downstream of each other in this pathway.

Altogether, these data indicate a strong collaboration between TREM2 and ApoE in several neuropathological hallmarks of LOAD. However, these discoveries raise new questions regarding the exact mechanism underlying this collaboration and the origin of ApoE involved in this partnership, which require further investigation. Moreover, these data reveal that a more exhaustive study of ApoE specifically in microglia is now necessary to better understand the link between AD and microglia.

### 8) TREM2: a brain teaser for therapeutic strategy in AD

Despite more than a century of research on AD, there is currently no treatment to prevent or cure the disease. Current treatments aiming to slow the progression of the disease target neurotransmission pathways altered in AD. The U.S. Food and Drug Administration has approved two types of medications that aim to slow down AD: cholinesterase inhibitors (Aricept, Exelon, Razadyne) and the NMDA receptor antagonist memantine (Namenda). However, the efficiency of these drugs has often been questioned (for review [163]). Although a considerable effort has been made to find new treatments, none have been met with any success yet in clinical trials.

While a great interest in TREM2 as a therapeutic target in AD is emerging, many impediments make its use potentially challenging. First, TREM2 risk variants are found in less than one percent of the population. In comparison, ApoE ε4-carriers represent 20% of the population [113, 164]. Although several studies suggest that, in certain conditions, targeting TREM2 could decrease AD-related pathologies as previously described, it is still unknown whether a potential TREM2-targeting treatment will be effective in non-carrier AD patients, which represent the majority of cases. Moreover, it remains to be determined whether a therapeutic strategy targeting TREM2 should activate or inhibit it. Indeed, as previously discussed, TREM2 can be either beneficial or pathological in AD depending on the disease models used, the context of the pathological insult, and the stages of pathology. To date, the best strategy seems to involve stimulating TREM2 signaling in the early stages of the disease, when amyloid deposition starts and before tau pathology and neuronal loss occurs. However, because of the dual role of TREM2 in AD, this hypothesis must be carefully tested to avoid worsening disease pathology. A similar strategy would be to stimulate TREM2 even before amyloid deposits form in the brain. Indeed, TREM2 deficiency prevents the transition of microglia to the MGnD phenotype, which prevents the beneficial effects of microglia on amyloid pathology [165]. Stimulating the microglial transition from homeostatic states to MGnD prior to amyloid accumulation may therefore delay the evolution of AD. Conversely, the observation that deletion of TREM2 in APPPS1-21 mice decreased the Aβ burden in 2-months-old animals but resulted in higher Aβ accumulation in the cortex of 8-months-old animals [131] suggests that MGnD could be beneficial in the later stages of amyloid pathology. This example highlights the complexity of targeting TREM2.

While the timing of when to stimulate TREM2 in order to treat AD pathology needs to be resolved, the question of how to target TREM2 also remains unaddressed. Immunotherapy using antibodies to stimulate TREM2 signaling are being developed by a number of groups. TREM2-activating antibodies have been tested *in vitro* and have been shown to induce activation of calcium and ERK signaling in human dendritic cells [32]. Such a strategy requires particular thoughtfulness because antibodies could alter binding of TREM2 ligands [75]. Modulating TREM2 expression or protein levels is another strategy of interest. Indeed, overexpression of TREM2 *in vitro* decreases inflammation and promotes phagocytosis [43]. *In vivo*, lentiviral approaches aiming to increase TREM2 expression in the brains of mice attenuated both cognitive and neuropathologic alterations [63]. However, it is important to note that lentiviral strategies cannot be used in humans because of the high risk of inducing oncogenic transformation.

Regardless of the strategy chosen to modulate TREM2 in AD, it is important to remember that microglia are not the only cells that express TREM2. Targeting of this receptor outside the brain will need to be assessed to make sure there are not unwanted side effects. Further, even in the brain, microglial functions are not limited to inflammatory responses (for review [166]) and modulating TREM2 signaling could induce several deleterious side effects that will have to be assessed. It seems obvious that the targeting of TREM2 in AD is a promising avenue to explore. However, there are clearly several obstacles that will need to be addressed before moving forward with such a strategy. The growing interest in TREM2, especially in the context of AD, may hopefully provide a better characterization of its roles and thus help to find a way around the possible barriers to its therapeutic targeting.

## Conclusion

**Fig. 2:**
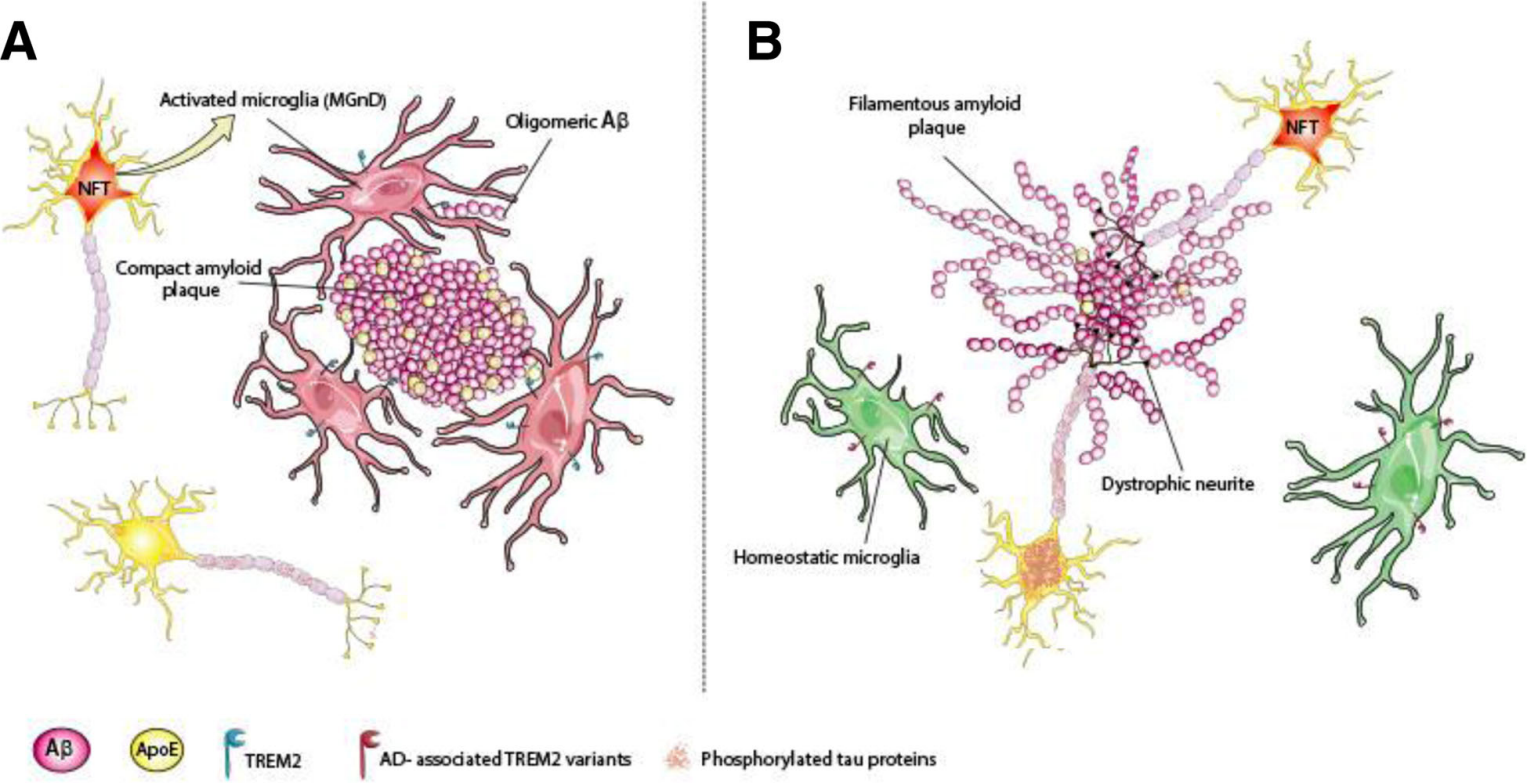
Schematic summary of the role of TREM2 and its variants in AD. **a**. Functional TREM2 has been suggested to allow microglia activation (by amyloid and NFTs for example), promote microglia clustering around plaques, amyloid uptake (early stage of the disease) and plaque compaction through binding to plaque-associated ApoE or directly to oligomeric Aβ. **b**. AD-associated TREM2 variants resulting in TREM2 partial loss-of-function abolished microglia clustering around plaque and phagocytic activity. These changes could be caused by a blockage of microglia in homeostatic stages because of less plaque-associated ApoE or other reasons. The consequences are filamentous plaques associate with increased dystrophic neurites and a possible increase of tau pathology (in early stages)

Despite a continually growing number of cases, AD is still under-characterized. In addition to the role of amyloid pathology, it is now clear that the pathogenesis of AD involves many alterations in the brain that interact synergistically, ultimately resulting in neuronal death. Beyond tau and amyloid pathologies, growing evidence suggests that neuroinflammation plays a crucial role in AD. Recent genetic studies (GWAS and whole genome sequencing) have confirmed this by identifying numerous genetic risk factors for AD associated with the immune system. Within these new genetic risk factors, a special interest has been directed at TREM2 in the last 5 years. Together, the data presented in this review strongly suggest an important role of TREM2 in AD at the level of amyloid and tau pathologies and inflammation, alone or in collaboration with other molecules such as ApoE (Table 1). Figure 2 illustrates current thinking and hypotheses regarding the role of TREM2 and its rare AD-associated variants in AD pathogenesis. Studies on TREM2 in the context of AD highlight its complexity. Indeed, *in vivo* studies suggest that TREM2 is injurious in the early stages of the disease and then becomes beneficial in the later stages. Finally, both ApoE and oligomeric forms of Aβ are able to bind and activate TREM2 making the elucidating of the TREM2 mechanism of action in AD difficult.

**Table 1:** Summary of the major findings on TREM2 in AD context

AD context	Major TREM2-AD related findings	Source	Citations
Risk factors	❖ Rare variants in TREM2 increase LOAD risk by 2- to 4- fold	AD patients	[104, 107–118]
Amyloid pathology	❖ Loss of functional TREM2 decreases microgliosis around plaques	5xFAD mice APPPS1-21 mice	[45, 75, 130, 132, 133]
	❖ Loss of functional TREM2 decreases plaque compaction	5xFAD mice APPPS1-21 mice	[75, 89, 90, 133]
Tau pathology	❖ TREM2 deletion decreases tau-mediated neurodegeneration	PS19 mice	[152, 153]
	❖ TREM2 deletion (1) or haploinsuficiency (2) increase tau pathology	hTau mice (1) PS19 mice (2)	[151] (1) [153] (2)
ApoE	❖ ApoE is a TREM2 ligand	*in vitro*	[76, 78, 79]
	❖ ApoE-induced switch from homeostatic to neurodegenerative microglia is TREM2-dependent	APPPS1-21 mice	[95]

Therefore, there are many outstanding questions that require further investigation. Most of the previously performed studies evaluated how TREM2 influences AD pathogenesis through partial or total deletion of TREM2. However, AD-related risk factors have also been associated with partial loss-of-function of TREM2. Consequently, further studies are needed to understand how these specific human risk variants affect AD. Furthermore, it is also necessary to understand the dual role of TREM2 in AD, which can reflect the often-reported dual role of neuroinflammation in this disease. In the same way, how TREM2 variants exactly alter AD remains unclear. Is it because of altered phagocytosis resulting in the accumulation of amyloid plaques and damaged neurons? Perhaps TREM2 variants increase the expression of pro-inflammatory molecules. Is TREM2 role in AD dependent of its interaction with ApoE? Is ApoE upstream or downstream of TREM2? It would be interesting to assess if similar TREM2 partial loss-of-function is observed in AD patients without AD-related TREM2 risk variants. This may occur through the interaction between TREM2 and APOE or oligomeric forms of Aβ, both of which are able to bind TREM2 and modulate its functions. Data obtained during the last 5 years has provided many answers regarding the role of TREM2 in AD, and has identified TREM2 as a therapeutic target. However, substantial questions regarding the potential targeting of TREM2 remain unclear. Is TREM2 beneficial or damaging at particular disease stages? The trigger of the disease? Or just a catalyst of an inevitable AD? The complexity of TREM2 in AD is beyond doubt and brings new questions with each new discovery. Addressing these questions first will be necessary to explain the role of TREM2 and microglia in AD and will help determine whether targeting it is a viable therapeutic strategy.

## Abbreviations

AAV: Adeno-associated virus
AD: Alzheimer’s disease
ApoE: Apolipoprotein E
Aβ: Amyloid-β peptide
ALS: Amyotrophic lateral sclerosis
CNS: Central nervous system
CSF: Cerebral spinal fluid
DAP: DNAX-activation protein
Fc: Fragment crystallizable
GSK-3β: Glycogen synthase kinase 3
GWAS: Genome wide association studies
HDL: High-density lipoproteins
ITAMs: Immunoreceptor tyrosine-based activation motifs
IL: Interleukin
IL-1β: Interleukin 1 beta
JNK: c-Jun N-terminal kinase
LDL: Low-density lipoproteins
LPS: Lipopolysaccharide
LOAD: Late-onset Alzheimer’s disease
LTA: Lipoteichoic acids
MAPK: Mitogen activated protein kinase
mTOR: Mammalian target of rapamycin
NOS2: NO synthase-2 transcription
NFT: Neurofibrillary tangle
PD: Parkinson’s disease
PI3K: Phosphatidylinositol 3-kinase
TLR: Toll-like receptor
TREM: Triggering receptors expressed on myeloid cells
TNF-α: Tumor necrosis factor alpha
